# Reference Intervals of Hematological Parameters Among Healthy Newborns in Eastern Sudan: A Hospital-Based Cross-Sectional Study

**DOI:** 10.3390/children13091244

**Published:** 2026-09-14

**Authors:** Alsir A. Abdallah, Nahla B. Mohamed, Enshrah M. Elamin, Hagir H. T. Ahmed, Ishag Adam

**Affiliations:** 1 Department of Obstetrics and Gynecology, New Halfa Hospital, New Halfa 31115, Sudan; alsirayes@gmail.com; 2 Department of Pathology, College of Medicine, Qassim University, Buraydah 51452, Saudi Arabia; nb.mohamed@qu.edu.sa (N.B.M.); en.ahmed@qu.edu.sa (E.M.E.); hag.ahmed@qu.edu.sa (H.H.T.A.); 3 Department of Obstetrics and Gynecology, College of Medicine, Qassim University, Buraydah 51452, Saudi Arabia

**Keywords:** hematological reference intervals, age, complete blood count, newborn, Sudan, sex-specific

## Abstract

**Highlights:**

**What are the main findings?**

• No statistically significant differences were found between male and female newborns for the evaluated hematological parameters.
• This justifies the pooling of data to establish a reference interval.

**What are the implications of the main findings?**

• The lower reference boundaries for hemoglobin, leukocytes, and platelets identified here differ markedly from Western-derived values.
• It is recommended to use these population-specific intervals, which may reduce inappropriate classification arising from the use of non-local reference intervals.

**Abstract:**

**Background:** Accurate interpretation of neonatal complete blood count (CBC) parameters is vital for managing life-threatening conditions in newborns. However, clinical decisions in Eastern Sudan frequently rely on non-local Western-derived or manufacturer-provided reference intervals (RIs), thereby risking diagnostic errors. Therefore, this study aimed to establish the local neonatal hematological RIs for healthy, term newborns in Eastern Sudan. **Methods:** This hospital-based, cross-sectional study was conducted at New Halfa Hospital. Umbilical cord blood samples were collected from 250 healthy term singleton neonates born to mothers with uncomplicated pregnancies and deliveries. Samples were analyzed using a Sysmex KX-21 hematology analyzer (Sysmex, Kobe, Japan). In accordance with Clinical and Laboratory Standards Institute (CLSI) guidelines, sex-specific 95% RIs were initially estimated, followed by assessment of whether sex-specific partitioning was warranted by calculating the central 2.5th and 97.5th percentiles. Differences in the distributions of hematological parameters between males and females were evaluated using the Mann–Whitney U test. **Results:** Of the total 250 newborns (123 males, 127 females), no statistically significant differences were found between male and female newborns for any of the eleven evaluated hematological parameters, justifying the pooling of data to establish unified RIs. The established 95% pooled RIs and medians were as follows: white blood cell (WBC) count: 6.33–20.90 × 10^3^/µL (median: 11.15 × 10^3^/µL); red blood cell (RBC) count: 2.91–5.04 × 10^6^/µL (median: 4.02 × 10^6^/µL); hemoglobin: 11.20–16.52 g/dL (median: 13.90 g/dL); hematocrit: 36.30–55.30% (median: 43.70%); mean cell volume (MCV): 93.41–127.53 fL (median: 109.70 fL); platelets: 49.20–438.68 × 10^3^/µL (median: 251.00 × 10^3^/µL); mean platelet volume (MPV): 7.36–11.55 fL (median: 9.30 fL); platelet distribution width (PDW): 16.10–19.37% (median: 16.90%). **Conclusions:** The results of this study provide preliminary locally derived neonatal CBC RIs for Eastern Sudan. The absence of statistically significant sex differences in this sample supports consideration of pooled neonatal RIs. The lower reference boundaries for hemoglobin, leukocytes, and platelets identified here differ markedly from Western-derived values and those reported in high-altitude cohorts, underscoring the critical clinical need to use these population-specific intervals, which may reduce inappropriate classification arising from the use of non-local reference intervals.

## 1. Introduction

The complete blood count (CBC) is one of the most commonly requested laboratory tests in neonatal medicine [1,2,3]. In the first days of life, accurate interpretation of CBC parameters is essential for diagnosing and managing critical conditions such as neonatal sepsis, polycythemia, hemolytic disease of the newborn, congenital cytopenias, and respiratory distress [2,3]. To guide clinical decision-making and avoid both dangerous diagnostic delays and unnecessary, invasive medical interventions, clinicians rely on established reference intervals (RIs) that describe the distribution of values observed in an appropriately selected reference population bounded by the 2.5th and 97.5th percentiles [4]. However, the widespread practice of applying international, Western-derived, or manufacturer-provided reference limits to local populations remains a significant source of diagnostic error in clinical settings [5].

This diagnostic mismatch is particularly acute in neonatology because newborn hematology is uniquely dynamic [6]. Immediately following birth, a neonate undergoes profound physiological adaptations as the infant transitions from the relatively hypoxic intrauterine environment to extrauterine life. This transition triggers a rapid contraction of plasma volume, a temporary suspension of erythropoietin production, and a gradual shift from fetal hemoglobin (HbF) to adult hemoglobin (HbA) [6]. Consequently, healthy newborns naturally exhibit macrocytic red blood cells, transient leukocytosis, and highly variable platelet dynamics that would be flagged as highly pathological in older pediatric or adult cohorts. Furthermore, neonatal blood parameters are significantly shaped by obstetric and maternal–fetal factors, including gestational age, mode of delivery, maternal nutrition, and the timing of umbilical cord clamping, which alters the volume of placental transfusion [7,8,9].

Beyond these universal physiological shifts, cumulative evidence shows that hematological profiles are heavily influenced by local genetic traits, ethnic backgrounds, regional nutritional baselines, and environmental exposures [3,5]. Across Sub-Saharan Africa, some populations of African ancestry have reported lower reference limits for selected hematological parameters than some Western reference populations [10,11]. This baseline difference was highlighted in West Africa by Adewumi et al. [12], who mapped reference values in Lagos, Nigeria, showing distinct hemoglobin and platelet profiles compared to Western standards. Interestingly, like the East African study by Angelo et al. [9] in Addis Ababa, the Nigerian cohort showed no significant sex-based differences in major CBC parameters at birth [12], suggesting that some neonatal biological patterns may transcend specific regional borders. Even so, relying on generalized external standards to evaluate local newborns risks widespread “diagnostic inflation,” leading to healthy infants being subjected to unnecessary sepsis workups, prolonged hospitalizations, or inappropriate exclusion from clinical trials.

While regional studies in neighboring East African nations have begun to address this gap, such as the study by Angelo et al. [9] in Addis Ababa, applying these external values directly across borders remains problematic. Neonatal hematology is highly population-specific and sensitive to environmental variables like altitude, which can significantly alter hemoglobin and red cell baselines compared to low-altitude populations. Furthermore, while the Ethiopian cohort demonstrated no significant differences between newborn sex except for red cell distribution width (RDW-CV) [9], such biological dynamics are highly cohort-dependent and must be locally validated. Consequently, even neighboring regional data cannot safely substitute for localized Sudanese RIs.

In Sudan, and particularly Eastern Sudan, the lack of localized neonatal reference standards is a pressing clinical challenge. Kassala State features a diverse, mixed demographic profile characterized by distinct migratory patterns, specific nutritional exposures, and a high regional burden of maternal infections [13,14]. This extreme regional vulnerability to hematological pathology is further underscored by Ahmed et al. [15], who reported that over 28% of pediatric admissions in neighboring Gadarif Hospital presented with anemia, with life-threatening cases frequently requiring emergency blood transfusions due to endemic sickle cell disease, malaria, and visceral leishmaniasis. Such a high regional burden of severe hematological illness makes establishing precise, localized reference ranges even more critical for ensuring early, accurate diagnosis.

While several studies have successfully mapped adult RIs in various Sudanese states [16,17,18,19], robust, statistically validated neonatal reference data remain virtually non-existent. A few localized neonatal studies have attempted to address this, but they are limited by narrow parameters or small sample sizes. Elgari and Waggiallah [7] investigated cord blood in Khartoum State, but they omitted critical CBC parameters such as RDW-CV, mean platelet volume (MPV), and platelet distribution width (PDW), restricting its diagnostic utility. Conversely, Muddathir et al. [8] established reference values in Kordofan State and identified significant sex-based differences in neonatal red cell counts, RDW, and MCHC, findings that contrast directly with the sex-neutral observations from Addis Ababa [9] and Lagos [12]. Importantly, this discrepancy may be methodological rather than biological (Muddathir et al.). The authors of [8] did not explicitly report testing their data for normality and relied on parametric Student’s *t*-tests, which can yield spurious statistical significance when applied to skewed neonatal hematological distributions. Furthermore, other regional work is severely underpowered; while Elgalil et al. [20] highlighted how maternal malaria alters Sudanese cord blood parameters (notably increasing RDW and reducing hemoglobin), their healthy control cohort was limited to only 30 uninfected subjects. These incomplete parameter profiles, contradictory regional findings, and underpowered healthy control groups may not meet the commonly recommended minimum sample size for non-parametric estimation within each reference partition that is recommended by the Clinical and Laboratory Standards Institute (CLSI) to calculate reliable RIs [21,22].

To bridge this critical gap in evidence, this study was designed to establish local CBC RIs from umbilical cord blood samples of healthy, full-term newborns delivered at New Halfa Maternity Hospital in Eastern Sudan. Using a powered cohort (*n* = 250) and non-parametric statistical methods recommended by the CLSI, this study aims to provide local pediatricians, neonatologists, and public health researchers with locally derived descriptive reference data.

## 2. Materials and Methods

### 2.1. Study Setting and Design

This hospital-based, cross-sectional study was conducted from November 2025 to January 2026 at the New Halfa Maternity Hospital, located in Eastern Sudan. New Halfa Maternity Hospital serves as the primary public referral facility for the New Halfa locality and its surrounding rural and peri-urban catchment areas, managing a high volume of obstetric cases and neonatal deliveries annually [14]. Data collection was carried out over an extended recruitment period to capture a representative seasonal and demographic distribution of the population. This study adhered strictly to the Strengthening the Reporting of Observational Studies in Epidemiology (STROBE) guidelines [23].

### 2.2. Study Population and Eligibility Criteria

Guided by previous similar studies [7,8,9], the study population comprised full-term newborns and their mothers who delivered at the facility during the study period.

Inclusion criteria: live-born, singleton, and with birth weight within 2500–4000 g, term neonates (gestational age ≥37–42 weeks, verified by maternal last menstrual period [LMP] or early gestational ultrasound) born to apparently healthy mothers with uncomplicated, uneventful pregnancies and deliveries, who provided informed written consent, were included.

Exclusion Criteria: multiple gestations (twins/triplets); women with chronic diseases such as diabetes mellitus, anemia, hypertension, preeclampsia, or thyroid disease; preterm infants (<37 weeks); smokers; neonates presenting with major congenital anomalies; babies with respiratory distress, meconium staining, gross congenital anomalies, or umbilical cords with true knots; and babies delivered by instrumental delivery were excluded.

### 2.3. Data Collection and Laboratory Procedures

Sociodemographic profiles and medical and obstetric history metrics were captured via structured, interviewer-administered questionnaires conducted with the mothers. Supplementary clinical and obstetric parameters, including residence, paternal education, maternal employment status, mode of delivery, newborn sex, newborn birth weight, and history of early pregnancy vaginal bleeding, were extracted directly from the hospital’s medical records.

Immediately after delivery, intrapartum management proceeded in accordance with standard institutional protocols. The umbilical cord was clamped at least 1 min after birth. Following umbilical cord clamping, the umbilical vein was promptly cannulated, and a 3 mL mixed umbilical cord blood sample was drawn into an ethylenediaminetetraacetic acid (EDTA) vacutainer. The samples were gently inverted and transported immediately to the central hospital laboratory. The CBC parameters, consisting of hemoglobin, red blood cell count (RBC), total white blood cell (WBC), platelet count, hematocrit, mean corpuscular volume (MCV), mean corpuscular hemoglobin (MCH), mean corpuscular hemoglobin concentration (MCHC), RDW-CV, MPV, and PDW, were determined using an automated hematology analyzer (Sysmex KX-21, Kobe, Japan) following the manufacturer’s protocols. To guarantee accurate and reproducible results, the analyzer underwent strict validation, calibration, and stabilization procedures prior to active testing to confirm compliance with clinical specifications. Stringent laboratory quality assurance was maintained throughout the study period via regular internal quality control runs and external validation procedures.

### 2.4. Sample Size Determination

Following the guidelines established by the CLSI [21,22], which recommend a minimum of 120 healthy reference individuals per partitioning group to estimate RIs, this study analyzed an expanded sample of 250 newborns (123 males and 127 females). This larger cohort was deliberately chosen to maximize statistical power, narrow the confidence intervals (CIs) around the 2.5th and 97.5th percentiles, and capture the biological variability inherent to this Eastern Sudanese population, thereby providing reliable reference limits for local clinical applications. To eliminate selection bias inherent in convenience sampling, a rigorous, systematic random sampling strategy (K = 3) was implemented. The sampling frame was constructed daily using the hospital’s labor room and maternity ward admission logs. Every third delivering mother who met the explicit inclusion criteria was selected sequentially until the target sample size was reached.

### 2.5. Statistical Analysis

Data management and statistical analyses were performed using the IBM Statistical Package for the Social Sciences (SPSS) for Windows, version 25.0 (SPSS Inc., Armonk, NY, USA). Categorical data and proportions were expressed as percentages (%). The outliers were handled via Tukey’s method [24]. For continuous variables, including age, parity, and all evaluated hematological parameters, normality was assessed using the Kolmogorov–Smirnov test. Because the continuous data exhibited a non-normal distribution, they are primarily reported as medians and interquartile ranges (IQRs), alongside means and standard deviations (SDs) for comprehensive descriptive reporting. To assess differences in median values between male and female newborns across hematological parameters, the nonparametric Mann–Whitney U test was applied. In strict accordance with CLSI recommendations for non-normally distributed reference data, the localized 95% RIs were determined non-parametrically by computing the central 2.5th and 97.5th percentiles of the distribution. Extreme boundaries are also reported using absolute minimum and maximum values. For all statistical tests, a two-sided *p*-value < 0.05 was defined as the threshold for statistical significance.

## 3. Results

### 3.1. Sociodemographic and Maternal Characteristics

In this study, a total of 250 healthy term mother–newborn pairs were enrolled. The median (IQR) maternal age was 25.0 (21.0–31.0) years, and the median (IQR) parity was 2 (1–4). Among the total studied mothers, 94.4% resided in urban areas. In terms of educational attainment, 78.8% of mothers had not completed secondary education. Among fathers, 67.2% had not completed secondary education. Regarding employment, the majority of the mothers were housewives (87.2%). Obstetric and neonatal delivery profiles indicated a nearly equal distribution in delivery mode: 48.8% (*n* = 122) of newborns were delivered vaginally, and 51.2% (*n* = 128) were delivered via Cesarean section. The sex distribution comprised 49.2% males and 50.8% females (Table 1). The median (IQR) for gestational age and birth weight was 38.0 (38.0–39.0) weeks and 3200 (2997–3412) g, respectively.

### 3.2. Complete Blood Count (CBC) Reference Intervals of Healthy Newborns

Table 2 presents the mean (SD), median (IQR), and 95% RIs (2.5th to 97.5th percentiles) for the CBC parameters among 250 healthy newborns. The parameters are reported for the overall cohort and stratified by sex (males vs. females). No statistically significant between-sex differences were observed at the prespecified 0.05 significance level for any of the analyzed hematological parameters.

### 3.3. White Blood Cell (WBC) Count

The overall median WBC count for the entire cohort was 11.15 × 10^3^/µL (IQR: 9.08–12.93 × 10^3^/µL), with a 95% RI of 6.33–20.90 × 10^3^/µL, with a median of 10.60 × 10^3^/µL (95% RI: 5.93–20.90 × 10^3^/µL) for males and a median of 11.30 × 10^3^/µL (95% RI: 6.44–22.08 × 10^3^/µL) for females. There was no statistically significant difference in WBC count between sexes (*p* = 0.127).

### 3.4. Red Blood Cell (RBC) and Hemoglobin Parameters

RBC indices showed highly consistent distributions between male and female newborns: Red Blood Cell Count: The overall median RBC was 4.02 × 10^6^/µL (95% RI: 2.91–5.04 × 10^6^/µL). No significant difference was observed between males and females (*p* = 0.453). Hemoglobin: The overall median hemoglobin concentration was 13.90 g/dL (95% RI: 11.20–16.52 g/dL). Values were virtually identical between males (median: 14.00 g/dL) and females (median: 13.70 g/dL; *p* = 0.183). Hematocrit: The overall median Hct was 43.70% (95% RI: 36.30–55.30%). Although males had a slightly higher median (44.20%) than females (43.20%), this difference was not statistically significant (*p* = 0.059). RDW-CV: The overall median RDW-CV was 15.70% (95% RI: 13.90–19.10%), with no significant difference between sexes (*p* = 0.068).

### 3.5. Red Blood Cell Indices (MCV, MCH, MCHC)

Erythrocyte indices remained stable across both sexes: MCV: The overall median MCV was 109.70 fL (95% RI: 93.41–127.53 fL), showing the characteristically macrocytic nature of neonatal red blood cells (*p* = 0.110 between sexes). MCH: The overall median MCH was 34.60 pg (95% RI: 28.64–38.85 pg; *p* = 0.579). MCHC: The overall median MCHC was 31.75 g/dL (95% RI: 27.44–34.70 g/dL; *p* = 0.168).

### 3.6. Platelet Parameters

Platelet counts and volumes showed wide ranges but no sex-specific variations: Platelet Count: The overall median platelet count was 251.00 × 10^3^/µL (95% RI: 49.20–438.68 × 10^3^/µL). The median platelet count was identical for males (251.00 × 10^3^/µL) and slightly higher in females (254.00 × 10^3^/µL), with no significant difference (*p* = 0.563). MPV: The overall median MPV was 9.30 fL (95% RI: 7.36–11.55 fL; *p* = 0.319). PDW: The overall median PDW was 16.90% (95% RI: 16.10–19.37%; *p* = 0.637).

## 4. Discussion

Although technical differences in specimen handling and analysis [25] and cord clamping time [26,27] among the different studies cannot be ruled out, standardized procedures were used as described in the Methods section to ensure quality. Population-level genetic, environmental, and nutritional differences could also contribute to variations among populations.

This hospital-based study is the first to establish neonatal CBC RIs in Eastern Sudan using a non-parametric approach consistent with CLSI recommendations [21]. By analyzing cord blood from a cohort of 250 healthy term newborns, full-term newborns in New Halfa, the authors have addressed a critical diagnostic gap. In clinical neonatology, using inappropriate reference standards risks “diagnostic inflation,” which can lead to healthy infants being subjected to unnecessary sepsis workups, prolonged hospitalizations, or inappropriate exclusions from clinical trials [5,9]. Our findings provide local pediatricians and neonatologists with locally derived descriptive reference data.

There were no significant differences (*p* > 0.05) between male and female newborns across all eleven evaluated hematological parameters. The absence of statistically significant between-sex differences supports consideration of pooled estimates to create unified, non-partitioned RIs for Eastern Sudanese neonates.

This finding aligns with the West African cohort in Lagos, Nigeria [12], and the East African cohort in Addis Ababa, Ethiopia [9], both of which reported no sex-based variations in major CBC parameters at birth. This consistency suggests that the physiological mechanisms regulating neonatal hematology immediately after birth, primarily driven by intrauterine hypoxia, placental transfusion, and rapid plasma volume shifts, are highly conserved biological processes that may be similar across some populations despite regional differences.

Conversely, our findings contrast directly with those of Muddathir et al. [8] in Kordofan State, Western Sudan; they reported significant sex-based differences in neonatal red cell counts, RDW, and MCHC. However, this discrepancy is likely methodological rather than biological. Muddathir et al. [8] did not explicitly report testing their data for normality and relied on parametric Student’s *t*-tests. When applied to highly skewed neonatal hematological distributions, parametric tests can violate core statistical assumptions, leading to spurious statistical significance [28,29]. In contrast, standard laboratory guidelines recommend utilizing non-parametric methods for reference data since biological parameters are rarely normally distributed [21,22]. By using the non-parametric Mann–Whitney U test on our verified non-normally distributed data, our analyses did not identify statistically significant between-sex differences; formal partitioning decisions should also consider clinical relevance and appropriate RI partitioning criteria. However, to determine whether a reference population needs to be separated into distinct subgroups (such as by sex or age) or if the data can be merged into a single reference range, laboratories rely on specific statistical and clinical guidelines. According to CLSI guidelines, the decision to partition or pool data is determined through an organized process combining statistical tests with clinical relevance [4].

### 4.1. Erythroid Parameters and Hemoglobin Profiles

Our cohort exhibited a median hemoglobin concentration of 13.90 g/dL and a median RBC count of 4.02 × 10^6^/µL. The erythrocyte indices showed a characteristically macrocytic profile, with a median MCV of 109.70 fL and a median MCH of 34.60 pg. These findings reflect the universal neonatal adaptation to the hypoxic intrauterine environment, which triggers elevated erythropoietin production and macrocytosis in utero [3].

The median hemoglobin (13.90 g/dL) in this study is lower than that reported in Khartoum State, Central Sudan (14.20 g/dL) [7], Kordofan State, Western Sudan (14.80 g/dL) [8], and Addis Ababa, Ethiopia (15.60 g/dL) [9]. The higher hemoglobin values observed in Addis Ababa (15.60 g/dL) are largely attributable to altitude, as high-altitude hypoxia naturally stimulates erythropoiesis [9]. However, the difference between our cohort and those in Khartoum and Kordofan is likely driven by regional maternal factors.

Kassala State has a high regional burden of maternal infections, endemic malaria, and specific nutritional exposures [13,14]. Maternal iron-deficiency anemia and subclinical placental malaria are known to restrict iron transfer across the placenta, resulting in lower neonatal iron stores and lower cord blood hemoglobin levels [20]. Given that Ahmed et al. [15] reported that over 28% of pediatric admissions at the neighboring Gadarif Hospital presented with severe anemia, our lower reference limit for hemoglobin (11.20 g/dL) provides a realistic, population-specific baseline that helps prevent the overdiagnosis of neonatal anemia in Eastern Sudan.

### 4.2. Leukocyte and Platelet Dynamics

The overall median WBC count in our study was 11.15 × 10^3^/µL. This central tendency is slightly lower than the medians reported in Khartoum (12.30 × 10^3^/µL) [7], Kordofan (11.74 × 10^3^/µL) [8], and Addis Ababa (12.40 × 10^3^/µL) [9].

Across Sub-Saharan Africa, healthy populations frequently exhibit lower baseline leukocyte and neutrophil counts than Western populations [10,11]. This variation is often attributed to genetic factors, such as the Duffy Antigen Receptor for Chemokines (DARC) gene polymorphism [11]. Our lower reference limit for WBC (6.33 × 10^3^/µL) is highly clinical; applying Western-derived neonatal standards (which often set the lower limit of normal WBC at 9.0 × 10^3^/µL or higher) to this population would misclassify many healthy Eastern Sudanese newborns as leukopenic or neutropenic, triggering unnecessary invasive evaluations for neonatal sepsis.

For platelet parameters, our cohort showed a median platelet count of 251.00 × 10^3^/µL, with a wide 95% RI of 49.20–438.68 × 10^3^/µL. The lower limit of this interval (49.20 × 10^3^/µL) is remarkably low compared to Western reference standards, which typically set the threshold for neonatal thrombocytopenia at 150.00 × 10^3^/µL [30,31]. However, highly variable platelet counts at birth are well-documented in African cohorts [12]. This wide distribution may be due to transient, benign platelet consumption during placental transfusion, as well as to subclinical maternal exposures endemic to Eastern Sudan [32,33,34]. Clinicians interpreting these findings should exercise caution. A platelet count falling below the conventional 150.00 × 10^3^/µL limit in this specific population should not automatically trigger aggressive, invasive diagnostic workups for thrombocytopenia unless accompanied by clinical symptoms of bleeding or other hematological abnormalities. In a large cohort, a few completely healthy infants will always fall into the extreme tails of a bell curve due to benign biological variation, remaining entirely asymptomatic [35].

The hematological values in different populations (Table 3) could, however, differ in sampling source, timing after delivery, cord-clamping practices, laboratory analyzers, eligibility criteria, and statistical methods across studies, which may substantially affect these comparisons [27,36].

We must highlight that our lower reference boundary for platelets (34.50 × 10^3^/µL) is exceptionally low. While transient neonatal platelet consumption during birth is documented, this lower limit is likely influenced by pre-analytical micro-clotting, a well-recognized challenge in umbilical cord blood sampling [37,38,39]. Clinicians should exercise extreme caution and repeat platelet counts via peripheral venipuncture if thrombocytopenia is suspected.

Importantly, our study successfully mapped essential platelet indices, including MPV and PDW, which were omitted in previous regional studies, such as the Khartoum State study [7]. These hematological parameters are vital for distinguishing between destructive and productive thrombocytopenia in neonatal intensive care units.

## 5. Conclusions

The results of this study could be used to establish the CLSI-compliant neonatal CBC RIs for Eastern Sudan. We demonstrated that sex-specific partitioning is unnecessary for these parameters at birth. The lower reference limits for hemoglobin, leukocytes, and platelets in our cohort differ from Western standards and high-altitude regional data. This highlights the importance of using localized RIs to prevent clinical errors. Therefore, we strongly recommend that healthcare facilities in Eastern Sudan adopt these newly established RIs rather than relying on external or manufacturer-provided limits. Further multicenter studies are needed to evaluate how these parameters change during the first week of life, particularly when using venous and capillary blood samples.

## Figures and Tables

**Table 1:** Sociodemographic characteristics of the studied mothers and their newborns at New Halfa Maternity Hospital, Eastern Sudan, 2026 (*n* = 250).

Variable		Median	Interquartile Range
Age, years		25.0	21.0–31.0
Parity		2	1–4
		Number	Percentage
Residence	Urban	236	94.4
	Rural	14	5.6
Maternal education level	<secondary	197	78.8
	≥secondary	53	21.2
Paternal education level	<secondary	168	67.2
	≥secondary	82	32.8
Maternal employment status	Housewife	218	87.2
	Employed	32	12.8
Mode of delivery	Vaginal	122	48.8
	Cesarean	128	51.2
Newborn sex	Male	123	49.2
	Female	127	50.8

**Table 2:** Descriptive statistics and reference intervals of complete blood count parameters by sex among healthy newborns in New Halfa, Eastern Sudan (*n* = 250).

Parameter	Sex	Mean (Standard Deviation)	Median (Interquartile Range)	95% Reference Interval (2.5–97.5%)	*p*-Value
**White Blood Cell Count (×10^3^/µL)**	Overall	11.57 (3.99)	11.15 (9.08–12.93)	6.33–20.90	0.127
	Males	11.18 (3.72)	10.60 (8.90–12.40)	5.93–20.90	
	Females	11.94 (4.22)	11.30 (9.40–13.70)	6.44–22.08	
**Red Blood Cell (×10^6^/µL)**	Overall	4.03 (0.54)	4.02 (3.71–4.36)	2.91–5.04	0.453
	Males	4.05 (0.52)	4.04 (3.75–4.38)	2.72–5.30	
	Females	4.02 (0.55)	3.99 (3.63–4.32)	2.90–4.93	
**Hemoglobin (g/dL)**	Overall	13.66 (1.61)	13.90 (12.78–14.70)	11.20–16.52	0.183
	Males	13.73 (1.84)	14.00 (13.00–14.90)	11.13–16.89	
	Females	13.59 (1.35)	13.70 (12.50–14.50)	11.20–16.12	
**Hematocrit (%)**	Overall	43.75 (5.24)	43.70 (40.38–47.00)	36.30–55.30	0.059
	Males	44.33 (5.50)	44.20 (40.60–47.80)	36.32–55.69	
	Females	43.18 (4.93)	43.20 (39.90–46.10)	36.30–54.98	
**Mean Cell Volume (fL)**	Overall	109.81 (9.09)	109.70 (105.20–113.73)	93.41–127.53	0.110
	Males	110.43 (9.79)	110.30 (105.80–114.90)	88.80–128.97	
	Females	109.20 (8.35)	108.50 (105.00–113.30)	94.98–126.06	
**Mean Cell Hemoglobin (pg)**	Overall	34.55 (2.83)	34.60 (33.40–35.90)	28.64–38.85	0.579
	Males	34.41 (2.56)	34.80 (33.40–35.70)	26.62–38.64	
	Females	34.67 (3.07)	34.40 (33.30–35.90)	30.26–39.22	
**Mean Cell Hemoglobin Concentration (g/dL)**	Overall	31.48 (2.05)	31.75 (30.58–32.60)	27.44–34.70	0.168
	Males	31.42 (1.74)	31.70 (30.30–32.40)	27.21–34.40	
	Females	31.53 (2.31)	31.90 (30.70–32.70)	27.84–34.96	
**Platelets (×10^3^/µL)**	Overall	243.64 (96.29)	251.00 (197.00–299.00)	49.20–438.68	0.563
	Males	244.12 (94.56)	251.00 (203.00–283.00)	49.28–539.00	
	Females	243.17 (98.32)	254.00 (189.00–309.00)	49.20–440.40	
**Platelet Distribution** **Width (%)**	Overall	17.04 (0.78)	16.90 (16.50–17.30)	16.10–19.37	0.637
	Males	17.00 (0.70)	16.90 (16.50–17.20)	16.10–18.70	
	Females	17.08 (0.85)	16.90 (16.50–17.30)	16.02–19.56	
**Mean Platelet Volume (fL)**	Overall	9.33 (1.37)	9.30 (8.60–9.80)	7.36–11.55	0.319
	Males	9.26 (1.29)	9.20 (8.60–9.80)	6.67–12.05	
	Females	9.40 (1.44)	9.30 (8.80–9.80)	7.52–11.22	
**Red Cell Distribution Width Coefficient of Variation (%)**	Overall	15.98 (1.35)	15.70 (15.00–16.73)	13.90–19.10	0.068
	Males	16.12 (1.35)	15.90 (15.10–16.90)	13.92–19.27	
	Females	15.85 (1.35)	15.60 (15.00–16.40)	13.74–19.10	

**Table 3:** Comparison of hematological reference intervals and central tendencies of the current Eastern Sudanese newborn cohort with regional studies from Sudan and other African countries.

		This Study (*n* = 250)			Khartoum, Sudan (*n* = 500) [7]	Kordofan, Sudan (*n* = 500) [8]	Nigeria (*n* = 130) [12]	Ethiopia (*n* = 139) [9]	
Parameter	Sex	Mean	Median	95% Reference Interval (2.5–97.5%)	Mean	Mean	Mean	Median	95% Reference Interval (2.5–97.5%)
White Blood Cell Count (×10^3^/µL)	Overall	11.57	11.15	6.33–20.90	12.3	11.74	-	12.4	6.6–19.4
	Males	11.18	10.60	5.93–20.90	-	11.65	13.16	12.6	6.6–19.3
	Females	11.94	11.30	6.44–22.08	-	11.83	13.07	12.4	6.1–23.2
Red Blood Cell (×10^6^/µL)	Overall	4.03	4.02	2.91–5.04	4.34	4.14	-	4.51	3.55–5.52
	Males	4.05	4.04	2.72–5.30	-	4.14	4.09	4.59	3.59–5.59
	Females	4.02	3.99	2.90–4.93	-	4.13	4.05	4.44	3.37–5.30
Hemoglobin (g/dL)	Overall	13.66	13.90	11.20–16.52	14.35	14.45	-	15.8	12.4–19.7
	Males	13.73	14.00	11.13–16.89	-	14.46	13.27	15.9	12.4–19.8
	Females	13.59	13.70	11.20–16.12	-	14.44	13.32	15.7	11.1–18.8
Hematocrit (%)	Overall	43.75	43.70	36.30–55.30	44.1	44.89	-	45.9	37.9–56.3
	Males	44.33	44.20	36.32–55.69	-	45.02	44.75	46.0	37.9–57.8
	Females	43.18	43.20	36.30–54.98	-	44.76	44.85	45.45	37.3–56.3
Mean Cell Volume (fL)	Overall	109.81	109.70	93.41–127.53	105.5	109.81	-	102.1	83.9–111.6
	Males	110.43	110.30	88.80–128.97	-	109.83	109.54	102.0	82.1–112.8
	Females	109.20	108.50	94.98–126.06	-	109.80	111.29	102.3	88.5–113.5
Mean Cell Hemoglobin (pg)	Overall	34.55	34.60	28.64–38.85	33.5	34.86	-	35.3	29.4–39.1
	Males	34.41	34.80	26.62–38.64	-	34.89	32.61	35.1	28.8–39.4
	Females	34.67	34.40	30.26–39.22	-	34.86	32.71	35.3	31.6–38.7
Mean Cell Hemoglobin Concentration (g/dL)	Overall	31.48	31.75	27.44–34.70	33.1	31.79	-	34.3	32.3–37.4
	Males	31.42	31.70	27.21–34.40	-	31.73	29.79	34.6	32.8–37.2
	Females	31.53	31.90	27.84–34.96	-	31.85	29.73	34.3	31.0–37.7
Platelets (×10^3^/µL)	Overall	243.64	251.00	49.20–438.68	256.0	257.15	-	236.0	146.0–438.0
	Males	244.12	251.00	49.28–539.00	-	250.0	223.64	230.0	146.7–466.2
	Females	243.17	254.00	49.20–440.40	-	263.0	226.69	241.5	146.0–418.2
Platelet Distribution Width (%)	Overall	17.04	16.90	16.10–19.37	-	-	-	11.0	9.1–15.7
	Males	17.00	16.90	16.10–18.70	-	-	-	11.2	9.3–16.2
	Females	17.08	16.90	16.02–19.56	-	-	-	10.8	8.6–14.8
Mean Platelet Volume (fL)	Overall	9.33	9.30	7.36–11.55	-	-	-	9.4	8.1–11.8
	Males	9.26	9.20	6.67–12.05	-	-	-	9.4	7.4–11.2
	Females	9.40	9.30	7.52–11.22	-	-	-	9.35	8.1–12.3
Red Cell Distribution Width Coefficient of Variation (%)	Overall	15.98	15.70	13.90–19.10	-	17.32	-	15.6	12.0–19.0
	Males	16.12	15.90	13.92–19.27	-	17.36	19.57	16.0	13.0–18.3
	Females	15.85	15.60	13.74–19.10	-	17.29	19.96	15.0	12.0–19.0

## Data Availability

The data presented in this study are available on request from the corresponding author due to privacy.

## Abbreviations

CBC	Complete Blood Count
CI	Confidence Interval
CLSI	Clinical and Laboratory Standards Institute
IQR	Interquartile Range
MCH	Mean Cell Hemoglobin
MCHC	Mean Cell Hemoglobin Concentration
MCV	Mean Cell Volume
MPV	Mean Platelet Volume
NICU	Neonatal Intensive Care Unit
PDW	Platelet Distribution Width
RBC	Red Blood Cell
RDW	Red Cell Distribution Width
RDW-CV	Red Cell Distribution Width Coefficient of Variation
RI	Reference Interval
SD	Standard Deviation
STROBE	Strengthening the Reporting of Observational Studies in Epidemiology
WBC	White Blood Cell
